# Social Support and Job Satisfaction in Kindergarten Teachers: The Mediating Role of Coping Styles

**DOI:** 10.3389/fpsyg.2022.809272

**Published:** 2022-03-14

**Authors:** Ying Yang, Xiaozhou Lu, Yongfei Ban, Ji Sun

**Affiliations:** School of Educational Sciences, Anshun University, Anshun, China

**Keywords:** job satisfaction, social support, coping styles, kindergarten teacher, mediation analysis

## Abstract

This study explored the relationship between social support and job satisfaction in kindergarten teachers, and the mediating role of coping styles in this relationship. A sample of 617 kindergarten teachers (*M*_*age*_ = 25.13 years, *SD* = 5.66 years) from kindergartens in mainland China completed questionnaires measuring social support, job satisfaction, and coping style. The results showed that social support was positively related to job satisfaction and positive coping style was positively associated with social support and job satisfaction. Furthermore, the results revealed that positive coping style, but not negative coping style, had a mediating effect on the relationship between kindergarten teachers’ social support and job satisfaction. This study provides initial evidence that coping style mediates the link between social support and job satisfaction in kindergarten teachers, and suggests that increasing social support and encouraging positive coping style could improve kindergarten teachers’ job satisfaction.

## Introduction

At present, kindergarten teachers generally face multiple responsibilities, heavy workloads, stressful working conditions, challenging behaviors, and diverse special needs of young children (Lambert et al., 2019), which greatly affects the job satisfaction of kindergarten teachers. Job satisfaction of kindergarten teachers not only affects their investment in teaching (Ma, 2019) but also influences the formation of healthy personalities in children (Hu et al., 2012). In addition, Toropova et al. (2021) noted that teacher job satisfaction contributes to not only the quality of teaching and learning but also the overall cohesion of the school, the professional status of teachers, and the wellbeing of teachers and their students. Furthermore, several studies have shown that higher job satisfaction in teachers reduces the likelihood of leaving their jobs (Blömeke et al., 2017; Yang et al., 2018), which is particularly important in times of high teacher turnover (Toropova et al., 2021). The frequent turnover of teachers not only undermines the educational process but also hinders the achievement of teaching objectives and the progress and development of students (Lambert et al., 2019). In recent years, the shortage of qualified teachers and the increase in teacher turnover have become a growing concern internationally (Ingersoll, 2017; European Commission, 2018). The high turnover of kindergarten teachers due to job dissatisfaction is an urgent problem in China especially (Xia et al., 2022). Therefore, kindergarten teachers’ job satisfaction has attracted widespread attention among educators, researchers, and policymakers (Wu et al., 2020). In summary, it is necessary to explore the potential predictors of kindergarten teachers’ job satisfaction to enhance job satisfaction and in turn, reduce turnover rates.

Job satisfaction refers to the pleasant or positive emotional state that an individual experiences as a result of an evaluation that promotes the achievement of personal job values (Locke, 1969). In teachers, Job satisfaction also reflects the emotional response to the teaching role (Zembylas and Papanastasiou, 2004). Kindergarten teachers’ job satisfaction involves cognitive evaluation and emotional feedback from kindergarten teachers on the work they carry out, and primarily includes satisfaction with the work itself, the work environment, treatment at work, and the management of the director (Lv, 2004). Most previous studies have focused on primary and secondary school teachers’ job satisfaction (Holsblat, 2014; Uzun and Özdem, 2017; Edinger and Edinger, 2018), whereas kindergarten teachers’ job satisfaction has received little attention. Lambert et al. (2019) argue that the more satisfied kindergarten teachers are with their jobs, the more effective they will be in supporting young children’s growth and development. Generally speaking, with greater support from parents, leaders, colleagues, friends, and families of young children, kindergarten teachers are more willing and confident and are better able to face and solve problems encountered during their work, and thus experience pleasure from successfully completing their work. Furthermore, Hassanein et al. (2021) stated that social support is one of the most stable and favorable predictors of personal satisfaction and wellbeing. Therefore, there appears to be a close relationship between kindergarten teachers’ social support, coping style, and job satisfaction. Importantly, however, limited research has been conducted to date on whether and how social support and coping styles affect kindergarten teachers’ job satisfaction. Thus, in the present study, we investigated the relationship between social support and job satisfaction in kindergarten teachers and identified potential mechanisms (i.e., coping styles) that mediate this relationship. This study will not only help to better understand the relationship between kindergarten teachers’ job satisfaction, social support, and coping styles and their mechanisms of action but also help kindergarten administrators to implement targeted measures to improve kindergarten teachers’ job satisfaction and in turn, improve the quality of preschool education.

### Social Support and Job Satisfaction in Kindergarten Teachers

Social support is defined as the information, emotional, and instrumental aid received by individuals from their social networks (Dunst et al., 1986). Social support can be intangible (such as emotional and verbal support) or tangible (such as goods and resources) (Kanu et al., 2008). Social support may come from friends, family, superiors, peers, and others (Zimet et al., 1988; Chiaburu and Harrison, 2008). According to Blau’s (1986) view on Social Exchange Theory (SET), social support plays a key role in promoting and predicting job satisfaction of employees (Ferguson et al., 2012; Zhang et al., 2015). When employees accept and perceive support from colleagues and leaders in the organization, they tend to respond by cultivating loyalty to the organization and demonstrating a liking for their work. Conversely, if employees do not receive the support described above, they may reduce their dependence on the organization and show dissatisfaction with their work (Lambert et al., 2016). The positive correlation between social support from others (e.g., organizational members) and Job satisfaction in primary and secondary school teachers has been confirmed by empirical studies (Holsblat, 2014; Uzun and Özdem, 2017). However, less is known about this relationship in kindergarten teachers. To our knowledge, only one study explored this relationship, and found that social support predicted job satisfaction of ethnic minority kindergarten teachers in China (Wu et al., 2020). In the current study, we recruited a sample of general kindergarten teachers to test this relationship, which enriches the research on job satisfaction in kindergarten teachers.

### Coping Styles as a Mediator

Although the relationship between social support and job satisfaction has been well established, the mechanism underlying this relationship is currently unclear; one potential mediator of this relationship in kindergarten teachers is coping styles. Coping is defined as the flexible, purposeful, and conscious adaptation of one’s own behavior to adapt to changes in the environment, that is, an individual’s conscious efforts in the face of pressure and external events (Compas et al., 2001). According to an individual’s attitude toward stress, coping styles can be positive or negative (Folkman and Lazarus, 1988; Liu, 2019). Koolhaas et al. (2010) proposed that different coping styles reflect changes in how individuals respond to stressors or challenging environments. Park and Adler (2003) believed that the two positive coping styles of problem solving and cognitive reconstruction are related to the overall positive satisfaction feedback of employees, while negative coping styles (such as trying to forget the whole thing) is often correlated with employees’ negative satisfaction (Armstrong-Stassen, 2004). Empirical study has also shown that coping styles is positively correlated with job satisfaction. For instance, the study by Liu et al. (2015) revealed that the active response of intensive care nurses of Chinese patients with heart disease significantly predicted their job satisfaction, and Wang (2018) found that the coping styles of Chinese employees significantly predicted their job satisfaction. Relatively few studies have been done on the relationship between coping styles and job satisfaction in the teaching domain. However, a study by Wang (2007) found that coping styles of rural secondary and elementary school teachers were significantly associated with life satisfaction, and positive coping styles such as problem solving tended to have a stronger effect on life satisfaction than negative coping styles such as self-blame. On the basis of prior study, we can hypothesize that kindergarten teachers’ coping styles is correlated with job satisfaction.

Furthermore, some research has found a significant positive correlation between social support and coping styles. For example, social support of adolescents (Chen et al., 2020), Hematopoietic stem cell transplantation caregiver (Luo et al., 2020) and parents of children with cleft lip (Yu et al., 2021) was significantly positively correlated with positive coping styles. The studies (Geng et al., 2018; Zhu et al., 2020; Tian et al., 2021) also found that the social support of Chinese medical staff and patients with lung cancer was positively correlated with coping styles and positively predicted positive coping styles. Shen and Ye (2004) showed that the increase in social support of secondary and elementary school teachers led to a stronger inclination to adopt adaptive coping style such as active awareness and direct action.

The above results show that social support, coping styles, and job satisfaction are closely related. Furthermore, it has been found that coping styles plays an important intermediary role in the relationship between environmental factors (organizational climate) and job satisfaction (Wang, 2007). Thus, coping styles may mediate the relationship between teachers’ social support and job satisfaction, despite the fact that few researchers have considered the mediating role of coping styles.

### Current Study

This study explored the correlation between social support, coping styles, and job satisfaction of kindergarten teachers. Moreover, we examined the relationship between kindergarten teachers’ social support and job satisfaction according to different coping styles (negative coping and positive coping) to determine which coping styles plays a more critical role in this relationship. This research sheds light on the problem-solving methods adopted by kindergarten teachers when facing problems, but could also help to improve job satisfaction in kindergarten teachers, thus maintaining and promoting their physical and mental health by extension.

On the basis of previous research, we made the following three hypotheses:

H1: Social support is positively associated with the positive coping styles of kindergarten teachers.
H2: A positive coping styles is positively associated with kindergarten teachers’ job satisfaction.
H3: Social support indirectly affects job satisfaction through positive coping styles rather than negative coping styles.

## Materials and Methods

### Participants

In November 2020, we recruited participants from a total of 20 kindergartens across five of the nine regions in Guizhou Province, China. First, five representative regions, Guiyang, Zunyi, Anshun, Qiannan, and Qiandongnan, were selected based on the differences in economic development; then, four kindergartens were randomly selected from each representative region using a random whole-group sampling method, and the questionnaire surveys were distributed to teachers in the selected kindergartens. A total of 680 kindergarten teachers participated in this study. However, 63 participants were excluded after data collection because of: (a) a regular pattern of responses; (b) significant missing data; and/or (3) contradictory responses to relevant items (e.g., inconsistent responses to homogeneous items or consistent responses to opposing items). Thus, 617 valid participants were included in subsequent analyses. We used the G*Power software ver. 3.1.9.7 (Faul et al., 2009) to estimate the sample size and found that a minimum of 319 participants would be needed to provide 95% power for a small to moderate correlation (*r* = 0.20, *two-tailed test* α = 0.05, 1-β = 0.95). Of these 617 kindergarten teachers, 28 (4.5%) were male and 589 (95.5%) were female; 205 (33.2%) participants had secondary vocational education (equivalent to high school), 264 (42.8%) had graduated from college, and 148 (24.0%) had a bachelor’s degree or above; 391 (63.4%) were from a private kindergarten and 226 (36.6%) were from a public kindergarten; 252 (40.8%) were married and 365 were unmarried (59.2%). The age of participants ranged from 17 to 53 years, with an average age of 25.13 ± 5.66 years.

### Measures

#### Multi-Dimensional Scale of Perceived Social Support

The MSPSS was developed by Zimet et al. (1988). The MSPSS includes 12 items (e.g., “I can talk about my problems with my family”) in three dimensions–family support, friend support, and significant other support (four items for each dimension). Each item is scored on a 7-point Likert scale (1 = very strongly disagree to 7 = very strongly agree). The Chinese version of the MSPSS was used for this study, which previous studies have shown to have a relatively high reliability and validity (Wang et al., 2017; Zhang et al., 2018). In our sample, the reliability coefficients for the total scale and the three subscales were 0.92, 0.80, 0.80, and 0.82, respectively.

#### Simplified Coping Styles Questionnaire

The Simplified Coping Style Questionnaire (Chinese version) was developed by Xie and Dai (2010) based on Folkman and Lazarus (1988) Coping Styles Questionnaire. The Simplified Coping Styles Questionnaire consists of 20 items in the two following dimensions: positive coping (12 items; e.g., “I seek advice from classmates, relatives or friends”) and negative coping (eight items; e.g., “I accept the reality, because there is no other way”). The items are scored on a 4-point scale from 3 (very often) to 0 (never).

A person’s overall coping tendency can be expressed as positive or negative depending on the standard score of positive coping styles minus that of negative coping styles. Coping tendency = standard score of positive CS (Z-score) –standard score of negative CS (Z-score) (Dai et al., 2010). A coping tendency score greater than zero indicates that the subject tends to adopt a positive coping styles under stress, and conversely, indicates that the subject tends to adopt a negative coping styles under stress (Nie et al., 2017). The scale has been widely used in coping styles research in China, has a good reliability and validity (Xie and Dai, 2010), and is supported by many published studies (Liu and Yang, 2019; Xie et al., 2020; Yao et al., 2021; Yu et al., 2021). In the current study, Cronbach’s α was 0.81 for the Simplified Coping Styles Questionnaire, and was 0.74 and 0.82 for positive coping and negative coping subscales, respectively.

#### Teacher Job Satisfaction Inventory

The Teacher Job Satisfaction Inventory was developed by Lv (2004). The Teacher Job Satisfaction Inventory consists of 30 items (e.g., “I think teaching is fun and I have a lot of fun with the kids”) in five subscales–working environment, work itself, further education and promotion, work remuneration, and school management. The items are scored on a 5-point scale from 5 (strongly agree) to 1 (strongly disagree). The higher the score, the higher the job satisfaction. In the scale, the total internal consistency coefficient is 0.87, the correlation coefficient between the subscale and the total scale is between 0.76– and 0.82, and the five subscales can explain 54.3% of the variance (Lv, 2004). The validity and reliability of the scale are also supported by related published studies (Li, 2011; Wang, 2014). In the present study, the computed Cronbach’s α was 0.85.

### Procedures

The questionnaire survey was conducted on-site in kindergartens by students majoring in preschool education at university, who were interning at kindergartens and had received training for administering the survey. Before the survey was carried out, participants were informed that participation in the survey was voluntary and anonymous, that they could withdraw from the survey at any time, that questionnaires should be completed independently, that the results of the survey were strictly confidential, and about how they should respond to the questionnaires. All participants provided written informed consent.

### Data Analysis

Firstly, descriptive statistics and Pearson’s correlation coefficients were performed for the social support, coping styles, and job satisfaction variables using SPSS 25.0. Secondly, we used multiple mediation analysis with bootstrap estimation (Preacher and Hayes, 2008) to test the significance of the mediating effects of coping styles (i.e., positive coping styles and negative coping styles) on the relationship between social support and job satisfaction.

In order to compare the strengths of the mediating effects of the different types of coping styles, we used the SPSS macro PROCESS program which was designed by Hayes (2018). The significance of the mediating effect was accepted if the 95% confidence interval (bias-corrected and accelerated; 5,000 bootstrap samples were specified) did not overlap with zero (Preacher and Hayes, 2008).

## Results

### The Relationship Between Kindergarten Teachers’ Social Support, Coping Styles, and Job Satisfaction

We used Pearson’s correlation coefficients to analyze the correlations between social support, coping styles, and job satisfaction in kindergarten teachers. The correlations between all factors are presented in Table 1. Social support was significantly positively associated with coping tendency (*r* = 0.500, *p* < 0.001), positive coping (*r* = 0.548, *p* < 0.001), as well as with job satisfaction (*r* = 0.442, *p* < 0.001). Additionally, job satisfaction was significantly positively associated with positive coping (*r* = 0.402, *p* < 0.001), as well as negative coping (*r* = 0.147, *p* < 0.001). However, social support was not significantly associated with negative coping (*r* = −0.045, *p* > 0.05).

**TABLE 1:** Descriptive statistics and correlations between the observed variables.

	M	SD	1	2	3	4
1 Social support	58.47	11.65	1			
2 Positive coping	0.00	1.00	0.55***	1		
3 Negative coping	0.00	1.00	–0.05	0.30***	1	
4 Coping tendency	0.00	1.19	0.50***	0.59***	−0.59***	1
5 Job satisfaction	97.16	12.68	0.44***	0.40***	0.15***	0.22***

***p < 0.001.

### Coping Styles as Mediators in the Relationship Between Social Support and Job Satisfaction

**FIGURE 1:**
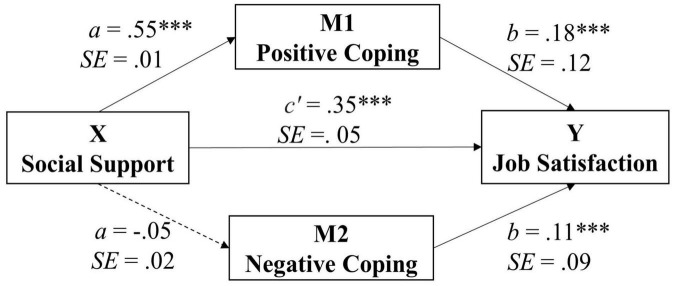
The mediation model for the effect of coping styles on the relationship between social support and job satisfaction. ***p < 0.001.

After identifying significant correlations between social support, coping styles, and job satisfaction, we conducted a mediation analysis with job satisfaction as the dependent variable, positive coping and negative coping as the mediators, and social support as the independent variable. The results are presented in Figure 1.

The results showed that: (1) social support was significantly associated with positive coping styles (SE = 0.01, β = 0.55, *t* = 16.27, *p* < 0.001, 95% CI [0.20, 0.25]); (2) positive coping styles was significantly associated with job satisfaction (SE = 0.12, β = 0.18, *t* = 3.90, *p* < 0.001, 95% CI [0.23, 0.71]); (3) social support was not significantly associated with negative coping styles (SE = 0.02, β = −0.05, *t* = −1.11, *p* = 0.267, 95% CI [−0.05, 0.02]); and (4) negative coping styles was significantly associated with job satisfaction (SE = 0.09, β = 0.11, *t* = 2.89, *p* = 0.004, 95% CI [0.09, 0.46]). Importantly, the total effect of social support on job satisfaction was 0.44 (SE = 0.04, *t* = 12.23, *p* < 0.001, 95% CI [0.41, 0.59]). The direct effect of social support on job satisfaction was 0.35 (SE = 0.05, *t* = 8.04, *p* < 0.001, 95% CI [0.29, 0.47]), whereas the indirect effect of social support on job satisfaction was 0.093 (SE = 0.03, 95% CI [0.04, 0.17]).

To further test the significance of the indirect effects of positive coping styles and negative coping styles, we used the bootstrap estimation procedure in the study. The estimated values of the mediating effects and their associated 95% confidence intervals are presented in Table 2. As shown in Table 2, social support had a significant indirect effect on job satisfaction *via* positive coping styles (indirect effect = 0.098, SE = 0.03, 95% CI ([0.05, 0.17]) and not *via* negative coping styles (indirect effect = −0.005, SE = 0.01, 95% CI ([−0.02, 0.00]) (see Figure 1 for the full mediation model). In other words, the results also suggested that positive coping styles rather than negative coping styles indirectly affected the relationship between social support and job satisfaction.

**TABLE 2:** Indirect effects and 95% confidence intervals.

Model pathways	Ind. Eff. Estimated	95%CI	
		Lower	Upper
Social support→Positive coping→Job satisfaction	0.098^a^	0.05	0.17
Social support→Negative coping→Job satisfaction	−0.005^a^	−0.02	0.00

*^a^Empirical 95% confidence interval does not overlap with zero. Ind. Eff. = Indirect effect.*

## Discussion

The purpose of this study was to explore the relationship between social support and kindergarten teachers’ job satisfaction, and whether social support had an indirect effect on job satisfaction through coping styles. The results showed that there was a significant positive correlation between kindergarten teachers’ social support, job satisfaction, and coping tendency. Importantly, kindergarten teachers’ social support had not only a direct effect on job satisfaction but also an indirect effect on job satisfaction through positive coping styles. The results of this study provide an empirical basis for improving kindergarten teachers’ job satisfaction.

As expected, social support of kindergarten teachers could significantly and positively predict job satisfaction, which is consistent with the results of previous studies (Yuh and Choi, 2017; Wu et al., 2020). The results showed that the more social support that kindergarten teachers received from family, friends, leaders, colleagues, the greater their satisfaction with their job. Teachers’ job satisfaction refers to teachers’ subjective feelings and experiences of the job itself, working conditions, and other aspects. kindergarten teachers’ work is characterized by heavy workloads, stressful working conditions, and considerable responsibility. Therefore, kindergarten teachers need active support from their families, kindergarten leaders, colleagues, and parents in order to carry out their work successfully. When kindergarten teachers receive external material or emotional support, this not only allows them to experience a sense of value and being understood and respected but also promotes positive emotional experiences of teachers’ willingness to engage in teaching and dedicate themselves to the teaching profession, which ultimately increases their job satisfaction. Thus, kindergarten teachers’ social support is closely related to job satisfaction. According to the SET (Blau, 1986), all human social interactions can be reduced to a form of exchange; that is, when people want to be rewarded for a social interaction, they tend to also pay a corresponding price. Thus, when employees receive social support from an organization, they may have a psychological sense of indebtedness to the organization, such that they actively participate in work to repay it, which contributes to greater job satisfaction (Colquitt et al., 2013). Brown (1996) also proposed that actively seeking social support from others in the workplace can enable employees to obtain positive emotional feedback or instrumental help, thus resulting in greater job satisfaction. A study by Pomaki et al. (2010) revealed that new teachers with higher levels of social support were less willing to leave their jobs when faced with higher workloads. The findings indicate that the focus of kindergarten administrators on enhancing kindergarten teachers’ social support is an effective way to not only increase their job satisfaction but also reduce kindergarten teachers’ tendency to leave the profession.

This study confirmed the significant indirect effect of social support on job satisfaction through positive coping styles, and further illustrated the mechanism by which kindergarten teachers’ social support affected their job satisfaction. Social support not only directly affected job satisfaction, but also indirectly affected job satisfaction through positive coping styles. These results indicate that being offered social support increased the levels of positive coping styles in kindergarten teachers, and high levels of positive coping styles increased job satisfaction. First, the finding that social support of kindergarten teachers was significantly and positively associated with positive coping styles supports H1. This is consistent with previous research on the relationship between social support and positive coping styles (Shen and Ye, 2004). In addition, social support was significantly and positively associated with positive coping styles, but not with negative coping styles, which is similar to previous research (Liu et al., 2015; Wang, 2018). This indicates that a higher the level of social support is more conducive to adopting positive coping styles in kindergarten teachers. Feeney and Collins (2015) “model of thriving through relational support” suggests that social support can provide supportive relationships for people who have experienced trauma, encourage them to challenge or expand themselves and find life goals, and seize every opportunity to verify their abilities. This will prompt the individual to adopt positive coping styles, such as facing the problem and working hard to solve the problem, when facing a stressful event.

In addition, our finding that the coping styles of kindergarten teachers is significantly and positively correlated with job satisfaction is similar to the previous finding (Zhou et al., 2013), which supports H2. Finally, we found that coping style was an important mediating variable in the relationship between social support and job satisfaction of the kindergarten teachers. Kindergarten teachers’ social support significantly and indirectly influenced their job satisfaction through positive coping styles rather than negative coping styles, which supports H3. This indicates that the higher level of social support of kindergarten teachers, the more likely they are to adopt positive coping styles to face and solve various problems encountered at work, which in turn increases job satisfaction. This further shows that social support and positive coping styles are not only factors affecting kindergarten teachers’ job satisfaction, but also protective factors for kindergarten teachers’ job satisfaction. A study by Yu et al. (2020) showed that increased social support and positive coping strategies significantly increase an individual’s sense of happiness and wellbeing. Therefore, the results of the study suggest that kindergarten teachers should proactively enhance their level of social support and actively face and solve problems encountered at work in order to increase their level of satisfaction with their jobs.

### Implications and Future Research

The results of the present study show that kindergarten teachers’ job satisfaction can be increased by establishing a good social support system and adopting more positive coping styles. Therefore, the results may inform kindergarten teachers to more effectively seek external support from various places (including workplaces) and through various social relations, as well as adopt more positive coping strategies, which will lead to an increase in job satisfaction. In addition, the results provide valuable insight for early childhood education stakeholders to work together to create a supportive social environment for kindergarten teachers. Moreover, our findings may help future studies investigating occupational attrition in kindergarten teachers in relation to social support, coping styles, and job satisfaction.

Although this study achieved the expected results, it has also some limitations that need to be overcome in our future research. First of all, we only used questionnaire survey method to collect data in this study. For further research, we should consider comprehensive use of data collection methods such as questionnaire surveys and interviews to reduce the possible deviations of a single method. Secondly, due to the limitations of time and space, we adopted a cross-sectional design in the study, which makes it difficult for us to clarify causality between variables. Future research could use experimental research or longitudinal research methods to further verify and extend the present results. Finally, under the mediation model, positive coping styles only partially mediated the relationship between social support and job satisfaction, which indicates that there are other mediators. Future research should explore other possible mediating factors, such as working environment, professional identity, emotional commitment, and self-efficacy, so as to more fully explain the mechanism underlying the effect of social support on job satisfaction, which could help to maintain and improve kindergarten teachers’ job satisfaction.

## Conclusion

To the best of our knowledge, this study is the first to examine the relationship between social support, coping styles, and job satisfaction in kindergarten teachers. This study investigated the correlations between social support, coping styles, and job satisfaction in kindergarten teachers, as well as the mediating effects of coping styles on the relationship between social support and job satisfaction. Social support and coping styles were positively associated with job satisfaction, and positive coping styles played a major mediating role in the link between kindergarten teachers’ social support and job satisfaction. These findings may provide new insights into improving job satisfaction in kindergarten teachers. Considering the findings of the present study, interventions aimed at increasing positive coping styles may be developed to boost job satisfaction among kindergarten teachers with low job satisfaction. In addition, because of the partial mediating role in the link between kindergarten teachers’ social support and job satisfaction, programs aiming at increasing social support may be developed to improve job satisfaction among such kindergarten teachers.

## Data Availability Statement

The raw data supporting the conclusions of this article will be made available by the authors, without undue reservation.

## Ethics Statement

The studies involving human participants were reviewed and approved by the School of Educational Sciences, Anshun University. The patients/participants provided their written informed consent to participate in this study.

## Author Contributions

YY and XL: research ideas and research design. YY, XL, YB, and JS: data collection and curation and project management, and fund acquisition. YY: data analysis and manuscript writing. All authors contributed to the article and approved the submitted version.

## Conflict of Interest

The authors declare that the research was conducted in the absence of any commercial or financial relationships that could be construed as a potential conflict of interest.

## Publisher’s Note

All claims expressed in this article are solely those of the authors and do not necessarily represent those of their affiliated organizations, or those of the publisher, the editors and the reviewers. Any product that may be evaluated in this article, or claim that may be made by its manufacturer, is not guaranteed or endorsed by the publisher.
